# Classification and Outcome Measures for Psoriatic Arthritis

**DOI:** 10.3389/fmed.2018.00246

**Published:** 2018-09-06

**Authors:** Ying Ying Leung, Alexis Ogdie, Ana-Maria Orbai, William Tillett, Laura C. Coates, Vibeke Strand, Philip Mease, Dafna D. Gladman

**Affiliations:** ^1^Department of Rheumatology and Immunology, Singapore General Hospital, Duke-NUS Medical School, Singapore, Singapore; ^2^Division of Rheumatology and Center for Clinical Epidemiology and Biostatistics, University of Pennsylvania, Philadelphia, PA, United States; ^3^Division of Rheumatology, Johns Hopkins University School of Medicine, Baltimore, MD, United States; ^4^Royal National Hospital for Rheumatic Diseases and Department of Pharmacy and Pharmacology, University of Bath, Bath, United Kingdom; ^5^Nuffield Department of Orthopedics, Rheumatology and Musculoskeletal Sciences, University of Oxford, Oxford, United Kingdom; ^6^Division of Immunology/Rheumatology, Stanford University, Palo Alto, CA, United States; ^7^Department of Rheumatology Research, Swedish Medical Center, University of Washington, Seattle, WA, United States; ^8^Division of Rheumatology and Krembil Research Institute, University Health Network, Toronto Western Hospital, University of Toronto, Toronto, ON, Canada

**Keywords:** psoriatic arthritis, outcome measures, core domains, core instruments, classification criteria

## Abstract

Psoriatic arthritis (PsA) is an inflammatory arthritis with multiple manifestations: peripheral/axial arthritis, enthesitis, dactylitis, psoriasis, and nail involvement. From having an agreed upon classification criteria in 2006, the assessment of PsA has advanced from uncertainties to development and validation of numerous specific outcome measures. The Group for Research and Assessment of Psoriasis and Psoriatic arthritis (GRAPPA) has spearheaded the development of a core domain set and is now working on a core outcome measurement set to standardize outcome measures for PsA, that will provide guidance for use of instruments in randomized controlled trials (RCTs) and longitudinal observational studies (LOS). This article summarizes and updates these work processes to improve assessment of this multisystem complex rheumatologic disease.

## Introduction

Psoriatic arthritis (PsA) is an inflammatory arthritis associated with psoriasis. It affects young adults of working age, with typical age of onset in the 30–50s (1). Destructive changes in bones can develop early resulting in joint damage and loss of function (2–4). Furthermore, higher inflammatory burden over time may lead to accelerated atherosclerosis, increased cardiovascular morbidity (5, 6), and possible early mortality for those with severe disease (7, 8). PsA is a unique disease entity that is different from other forms of chronic inflammatory arthritis, in terms of clinical manifestations, pathogenesis, response to treatment, and prognosis (9). Thus, clinicians and rheumatologists need to be aware of the classification and assessment of PsA to optimize care for these patients. In this article, we aim to summarize the development of classification criteria and outcome measures in PsA.

### Classification criteria

An important aspect of studying a “disease entity” is whether one can identify it as sufficiently homogenous to be distinct from other conditions. Classification criteria serve to ensure that patients recruited into RCTs have the same “disease,” so that results of these trials can be accurately interpreted (10, 11), but they are not designed for diagnostic purposes.

Before 2006, there were no validated case definitions or universally agreed upon classification criteria for PsA. Most historical studies used the case descriptive definition proposed by Moll and Wright (12), which defined PsA as an inflammatory arthritis in the presence of psoriasis and usually the absence of rheumatoid factor (RF). Subsequent cohorts from different centers identified different proportions of PsA patients divided into asymmetrical oligoarthritis and symmetrical polyarthritis subgroups (13, 14), possibly because of inclusion of differing proportions of RF negative rheumatoid arthritis (RA) patients based on the Moll and Wright case definition (13). Several classification criteria have subsequently been proposed (15). Table 1 summarizes the pros and cons of the various classification criteria sets for PsA. A comparative study using retrospective cross sectional and prospective multi-center datasets found high specificities (>90%), and variable sensitivities (42–98%) differentiating PsA from RA across these different classification criteria sets (15). In addition, the performance of different criteria sets distinguishing PsA from other arthritides has not been tested.

**Table 1:** Operational definition of classification of psoriatic arthritis.

**Criteria**	**Details**	**Pros and Cons**
CASPAR 16	Inflammatory articular disease (joint, spine, or entheseal) AND ≥3 points from the following: •Evidence of psoriasis - Current psoriasis (scores 2 points) or - Personal history of psoriasis or - Family history of psoriasis •Psoriatic nail dystrophy •A negative test for rheumatoid factor •Dactylitis: - Current dactylitis - History of dactylitis •Radiological evidence of juxta-articular new bone formation	Pros: •Developed via international collaborative cohort of PsA experts •Easy to use •Good sensitivity and specificity •Allowing PsA to be classified without psoriasis when other features are present •Allowing patients with positive RF to be classified •Validated in established and early PsA cohorts, and across multiple ethnicities •Has gained acceptance and adoption over time •The most commonly adopted classification in modern randomized controlled trials (RCTs) and longitudinal observational studies (LOS) Cons: •Inflammatory articular disease in joint, spine or enthesis are not well-defined, could be challenging to use in non-rheumatology settings
Moll and Wright 12	Arthritis AND Psoriasis AND NOT Positive Rheumatoid Factor (RF)	Pros: •The original diagnostic criteria for PsA •The simplest and the most frequently used historically Cons: •May have used implicit, but undeclared, features for classification, resulting in later cohorts classifying patients with different features •Excluded patients with positive RF •Must have psoriasis to be classified •The original proposed five subgroups of PsA are not sustained over time and treatment (Eg polyarthritis vs. oligoarthritis)
ESSG 17	Synovitis or inflammatory spinal pain AND Psoriasis or personal history of psoriasis	Pros: •Easy to use •Allows PsA to be classified without current psoriasis Cons: •Main purpose of development was to classify Spondyloarthopathies as a single entity •Lower sensitivity
Vasey and Espinoza 18	Psoriasis or psoriatic nail lesion AND Peripheral pattern ^α^ or Central pattern^β^ *α: >4 weeks arthritis of DIPJ; or asymmetrical peripheral arthritis (included sausage digit); absent RF or rheumatoid nodule; or radiographic changes (Pencil-in-cup deformity, whittling of terminal phalanges, fluffy periostitis, and bony ankylosis)* *β: >4 weeks Spinal pain and stiffness with the restriction of motion; or Grade 2 symmetric sacroiliitis, or Grade 3 or 4 unilateral sacroiliitis according to the New York criteria*	Pros: •Easy to use •Only describes two patterns of PsA Cons: •Must have psoriasis or nail lesions to be classified •Radiographic changes just classify late disease •Very few validation studies •Has not been used in RCTs/LOS

*ESSG, European Spondyloarthritis Study Group; CASPAR, ClASsification criteria for Psoriatic Arthritis; PsA, psoriatic arthritis; RCT, randomized controlled trials; LOS, longitudinal observation studies; RF, rheumatoid factor*.

The ClASsification criteria for Psoriatic Arthritis (CASPAR) study group was established to derive new data driven classification criteria for PsA (19). The study group subsequently formed the Group for Research and Assessment in Psoriasis and Psoriatic Arthritis (GRAPPA) including rheumatologists, dermatologists, patients and others, that, with Outcome Measures in Rheumatology (OMERACT) have pioneered the work to establish the best outcome measures for PsA. The CASPAR study group collected data prospectively from 32 centers worldwide, 588 consecutive PsA patients and the next 536 patients seen with inflammatory arthritis as the control (72% RA). PsA cases and controls were classified by existing criteria for respective accuracy, and new classification items were constructed. The CASPAR criteria (Table 1) include characteristic dermatologic, clinical, and radiographic features and have demonstrated high sensitivity (91.4%) and specificity (98.7%) (16). The CASPAR criteria enable classification of PsA in patients without psoriasis, but other associated features. The CASPAR criteria have subsequently been validated in early PsA cohorts (20, 21), retrospective cohorts (22), primary health care settings (20), and other ethnicities (23). Currently, the CASPAR criteria have become the most widely used criteria for recruitment in both RCTs and LOS (14). The only concern about the CASPAR criteria is the initial qualification criterion (stem question): inflammatory musculoskeletal (MSK) disease including either spinal, peripheral joint or entheseal manifestations. It may be difficult for practitioners other than rheumatologists, such as dermatologists, to differentiate inflammatory arthritis from other non-specific aches and pains in tendons and joints. GRAPPA is currently working on methods to better define inflammatory MSK disease (24).

More recently, the Assessment of SpondyloArthritis International Society (ASAS) developed peripheral (pSpA) and axial spondyloarthritis (AxSpA) criteria, where PsA could be classified under both pSpA or AxSpA (25). In a cohort of early arthritis, the pSpA criteria were found to have lower sensitivity for early PsA compared with the CASPAR criteria (26). However, patients with dactylitis or enthesitis or predominantly AxSpA were excluded from this validation cohort which may have limited the performance of these criteria. Moreover, all validation studies for spondyloarthritis classification criteria thus far have been based on a case control design; introducing possible bias by over-estimating sensitivity and specificity of the criteria (27). After all, constructing criteria for SpA may be challenging because it is inclusive of many heterogeneous disorders. It may be more rational to separate PsA and AxSpA into different more homogeneous phenotypic entities, thus facilitating both instrument development and measurement of clinical outcomes in the long term (28, 29).

### Measurement of meaningful outcomes in *PsA*

Advances in the development of biologic therapies have offered hope for better treatment for patients with PsA since early 2000s. However, meta-analyses of results from RCTs have been hampered by the lack of homogeneity in outcome measures in PsA. Just a decade ago, most of the instruments for PsA were borrowed from RA RCTs (30, 31). Instruments that function well in other forms of arthritis may not necessarily measure what it is intended to be measured in PsA. For instance, the reduced 28-joint count in RA that focuses on hand joints grossly underestimates disease burden in PsA as the feet are most commonly affected in PsA (32). Unlike measurement of blood sugar levels in diabetes mellitus, the concept of disease control in PsA is a construct that is more difficult to define. It is generally accepted that amelioration of inflammation with effective treatment reduces symptoms, prevents damage accumulation and reduces adverse health outcomes from comorbidities. Traditional serum inflammatory biomarkers such as erythrocyte sedimentation rate and C-reactive protein are well-known to be elevated in < 30% of patients with active disease (33), and therefore may not reflect underlying disease activity. A challenge to quantify disease activity and impact of PsA are the diverse clinical manifestations that span across peripheral and axial joint arthritis, enthesitis, dactylitis, psoriatic skin, and nail lesions. Moreover, while disease activity and disease impact in PsA are different constructs they are not totally independent of each other. People affected by PsA may have different views compared with clinicians on which disease manifestations and impact are important to them (34).

Over the past decade, many disease specific instruments for the assessment of various domains have been developed and validated for use in RCTs (35). However, the lack of standardization of domains and instruments in RCTs is problematic. Heterogeneity in domain measurement with multiple instruments per domain in PsA RCTs (36, 37) can hinder the comparability of efficacy assessments across interventions.

Established in 1992, the OMERACT international consensus effort has been working to improve outcome measures for use in RCTs and LOS in rheumatology, building on “Truth,” “Discrimination,” and “Feasibility” (38). “Truth” means measuring what is intended to be measured in a relevant and unbiased manner. It captures issues of face, content, construct, and criterion validity. “Discrimination” means whether an instrument discriminate between situations of interest, such as disease states at different time points to measure change and also captures issues of reliability and sensitivity to change. “Feasibility” assesses whether the instrument can be easily applied, given constraints of time, money, and interpretability.

OMERACT has recently updated and outlined a conceptual framework for core set development (Filter 2.1) that encompasses both patient-centered and intervention specific information (39). This framework specifies four key components termed “Area” of a health condition to ensure comprehensive coverage: three Areas that describe the “Impact of Health Conditions,” specifically Death, Life Impact, and Resource Use; and the fourth Area that describes Pathophysiological manifestations. The OMERACT filter 2.1 has also attempted to distinguish two major components in outcome research, namely, determining “what to measure” before deciding on “how to measure” them. Within this concept, the development of “Core Domain Sets” followed by “Core Measurement Sets” with defined instruments was set forth. Each instrument in the final core measurement set must prove to be truthful, discriminative, and feasible (39).

### Development of core domain set for *PsA*

Working with OMERACT, GRAPPA researchers defined the first core domain set for use in both RCTs and LOS in 2006 (40). A need to update the core domain set was identified as our understanding of PsA advanced over the years, and to include important patient input. The concept of involving patients as research partners (PRPs) was recognized as an essential and valuable component in the process (39, 41). Without incorporating the experience of disease through involvement of patients it is not possible to fulfill the “truth” or “feasibility” element of the OMERACT filter. The role of PRPs has evolved from informing disease impact via participation in qualitative studies to much broader participation in study design and conduct (42). OMERACT has continued to publish updated guidelines on core set development. Therefore, it was deemed important to update the PsA core domain set incorporating patients' perspectives in multiple steps, and in accordance with the OMERACT filter 2.1 (43).

**Figure 1:**
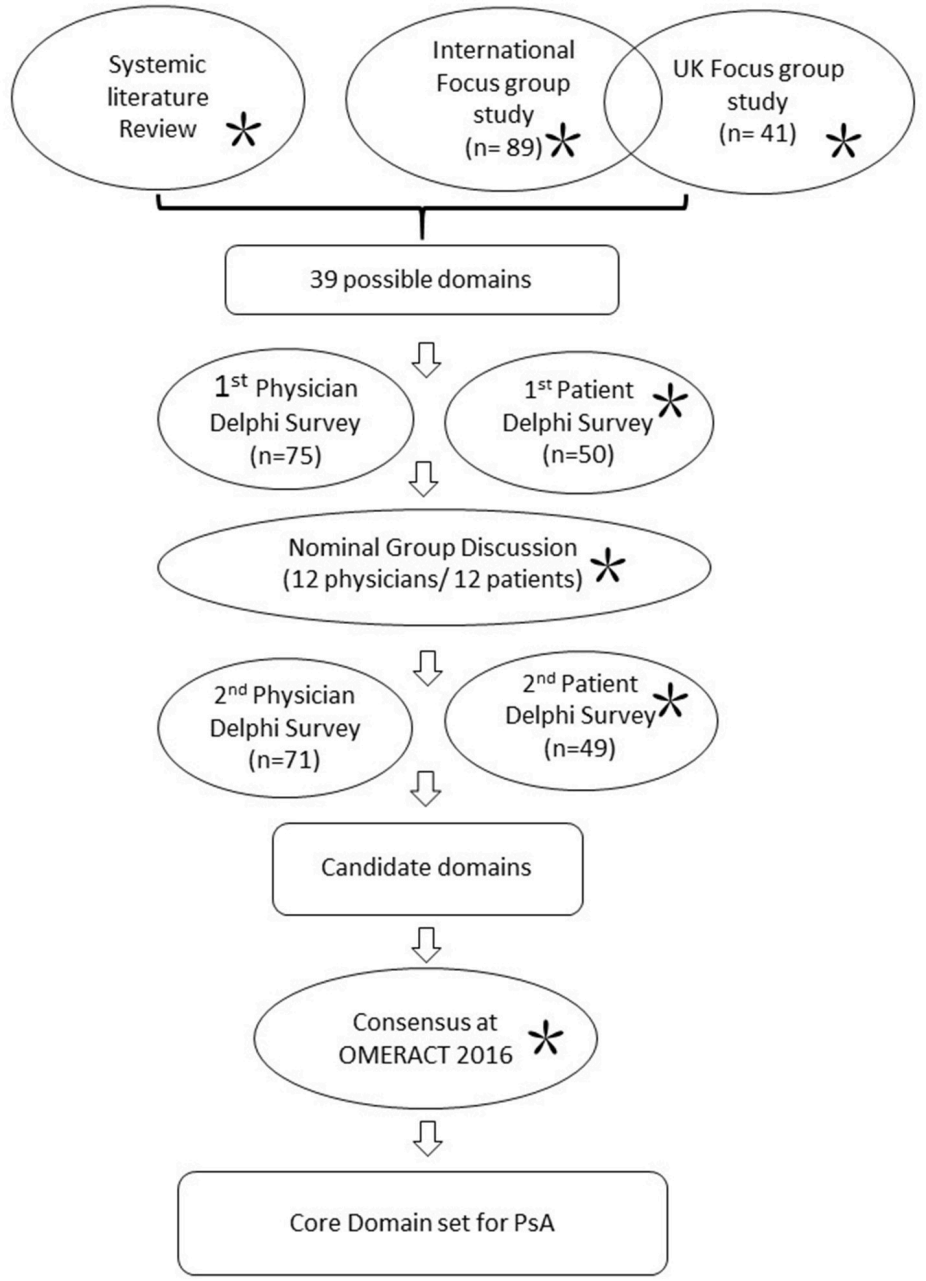
Work stream for update of core domain set for PsA 2016. Work processes in circles. *Indicates involvement of patients.

GRAPPA has assembled an international collaborative effort to update the PsA core domain set since 2014. Patients have been involved in all steps of development (conducting focus groups and analyzing data, including PRPs who functioned in the high-level conduct of the research; Figure 1). The detailed processes of core set development have been published elsewhere (44, 45). In brief, it started with identification of possible domains from a comprehensive literature search, as well as the previously identified domains from 2006 (37). Patients' perspectives on how PsA impacts their lives were actively sought through an international focus group involving 130 patients across 7 countries representing 5 continents; together with a UK multi-centered focus group study. Two independent Delphi surveys with health care providers and patients were then conducted to rate the importance of each domain. The selection of each domain was discussed in face-to-face nominal groups conducted with 12 clinicians, 12 patients, and 2 non-voting fellows in March 2016 in New Jersey, USA. Thorough exchange of ideas and perspectives between clinicians and patients were achieved, revealing differences but aiding resolution and consensus. A second Delphi survey round followed. Candidate domains were presented and endorsed at OMERACT 2016.

**Figure 2:**
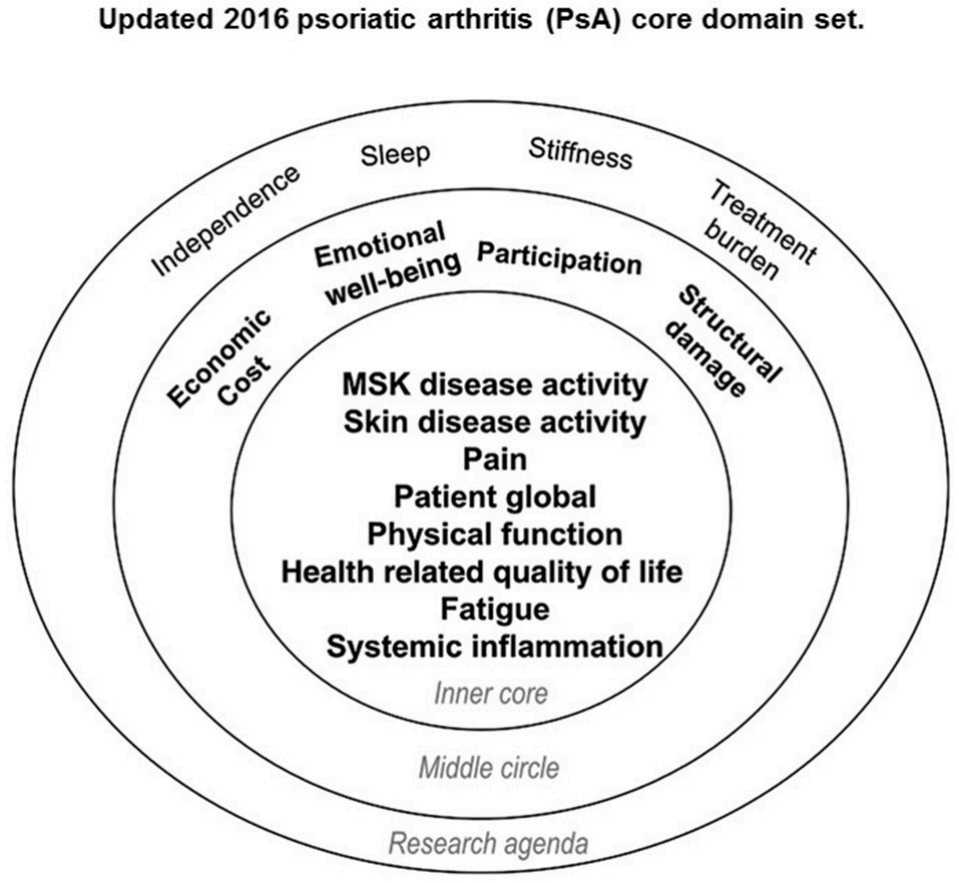
Updated 2016 psoriatic arthritis (PsA) core domain set. Reproduced with permission from Orbai et al. (45). Musculoskeletal (MSK) disease activity includes peripheral joints, enthesitis, dactylitis, and spine symptoms; skin activity includes skin and nails; patient global is defined as patient-reported disease-related health status. The inner circle (core) includes domains that should be measured in all randomized controlled trials and longitudinal observational studies. The middle circle includes domains that are important but may not be feasible to assess in all RCTs and LOS. The outer circle (or research agenda) includes domains that may be important but need further study.

The final PsA core domain set includes three parts: an inner circle (should be measured in all RCTs and LOS), a middle circle (important to be measured at some point in the drug development program, but not mandatory), and the outer circle that represents the research agenda (Figure 2). The inner circle includes MSK disease activity (peripheral joints, enthesitis, dactylitis, and spine involvement), skin disease activity (skin and nails involvement), pain, patient's global assessment, physical function, health-related quality of life, fatigue, and systemic inflammation biomarkers (Figure 2). This core domain set, developed with extensive patient involvement and representation of stakeholders from 5 continents, achieved general consensus and provides guidance as to what to measure in PsA RCTs and LOS.

### Development of core outcome measurement set for *PsA*

Following development of the core domain set, the GRAPPA-OMERACT PsA core set working group is leading the work to standardize the core outcome measurement set according to the OMERACT filter methodology (46). This methodology evaluates each instrument with four pillars of OMERACT:

1. Whether an instrument is perceived as a match to the domain intended to measure by stakeholders (Domain match)
2. How feasible is the instrument to be used (Feasibility).
3. How truthful numerically it is matching to the domain or construct (Truth)
4. How responsive the instrument is to change of status of disease (Discrimination).

The work stream started with evaluation of the existing evidence on PsA instrument properties through systemic literature reviews and data analyses from RCTs, followed by Delphi processes with stakeholders (including patients, clinicians, methodologists, and payers), and working group meetings and discussion (46). Thus far, evaluation of evidence on existing patient reported outcomes (47), MSK disease activities and systemic inflammation (48) have been completed. The 66/68 joint count and Psoriatic arthritis impact of disease (PsAID12) that measure peripheral MSK disease activity and health-related quality of life or impact of disease, respectively, have been thoroughly evaluated by the GRAPPA/OMERACT working group. At the OMERACT 2018 conference in Terrigal Australia, the 66/68 joint count was endorsed and PsAID received provisional endorsement.

The GRAPPA core set working group will continue its work on proper evaluation of measurement properties for existing and new instruments for other domains and will seek consensus to standardize the outcome measurement set for the other domains, including MSK manifestations such as dactylitis and enthesitis; and physical function will be taken forward for appraisal using the OMERACT filter 2.1 (Table 2). The aim is to create a full core instrument set to complete the core domain set for PsA in the coming years.

**Table 2:** Examples of candidate instruments for the *PsA* core instrument set.

**2016 PsA Core outcome set (inner circle)**	**Candidate outcome measurement instruments prioritized for apprasial using OMERACT filter 2.1**
**MSK DISEASE ACTIVITY**	
MSK disease activity/Arthritis	66/68 swollen/tender joint count^*^^†^
MSK disease activity/Dactylitis	Leeds Dactylitis Index (LDI) (0–60) LDI basic, no grading (score range 0–20)
MSK disease activity/Enthesitis	Leeds Enthesitis Index (LEI)—(6 sites) Spondyloarthritis Research Consortium of Canada Index (SPARCC)—(16 sites) Maastricht Ankylosing Spondylitis Enthesis Score (MASES)—(13 sites) Impact Index—(4 sites)
MSK disease activity/Spine	Research agenda
**SKIN DISEASE ACTIVITY**	
Skin disease activity/Skin	Psoriasis Area and Severity Index (PASI) Psoriasis Body Surface Area (BSA) Target psoriatic skin lesion score (0–12) Physician global assessment of psoriasis (PSGA/PGA) (0–5) Mean Body Surface Area involved ***PROMs*** Psoriasis Symptom Inventory (PSI) Psoriasis Symptom Diary (PSD) Worst Itch VAS Patient Assessment Skin Status (Likert)
Skin disease activity/Nail	***Physician performed by inspection of (looking at) the patient's nails*** Nail Psoriasis Severity Index (NAPSI) (0–80 finger nails only; or 0–160 finger and toe nails) Modified Nail Psoriasis Severity Index (mNAPSI) (0–130) Target NAPSI score (0–13) VAS Nail Psoriasis
**PAIN**	
	***PROMs*** 0–100 VAS Pain (1 week recall) 0–100 VAS Pain (recall not specified) 0–10 NRS Pain (1 week recall) PROMIS Pain Intensity PROMIS Pain Interference
**PATIENT GLOBAL**	
	***PROMs:*** *Patient global due to psoriasis* 0–10 NRS (1 week recall) 0–100 VAS (1 week recall) *Patient global due to arthritis* 0–10 NRS (1 week recall) 0–10 NRS (1 day recall) 0–100 VAS (1 week recall) *Patient global due to skin disease* 0–10 NRS (1 week recall) 0–100 VAS (1 week recall) 0–100 VAS (recall not specified)
**PHYSICAL FUNCTION**	
	Health Assessment Questionnaire Disability Index (HAQ-DI) SF-36 Physical Function domain PROMIS Physical Function
**HRQoL/LIFE IMPACT**	
HRQoL	***PROMs*** *Disease specific:* PsAID^*^^†^ *Generic:* SF36 PCS/MCS and 8 domains *Generic:* PROMIS Profiles *Generic to Dermatology (not specific to psoriasis):* Dernatology Life Quality Index (DLQI)
**FATIGUE**	
	***PROMs*** Functional Assessment of Chronic Illness Therapy (FACIT)-Fatigue SF-36 Vitality domain PROMIS Fatigue VAS Fatigue NRS Fatigue Fatigue Severity Score (FSS) Fatigue Assessment Scale (FAS) Bristol RA Fatigue (BRAF) (3 NRS scales) Multidimensional Assessment of Fatigue (MAF)
**SYSTEMIC INFLAMMATION**	
	***Laboratory assays from a patient's blood/serum*** C-Reactive Protein (CRP)^*^ Erytrocyte Sedimentation Rate (ESR)^*^

* *Completed evaluation*;

† *endorsed/ provisionally endorsed; PROM, patient reported outcome measures; VAS, visual analogue scale; NRS, numeric rating scale; MSK, musculoskeletal; PROMIS, Patient-Reported Outcomes Measurement Information System; SF-36, Medical Outcome Short Form 36; PCS, Physical Summary Scale of SF-36; MCS, Mental Summary Scale of SF-36; PsAID, Psoriatic Arthritis Impact of Disease*.

## Conclusion

With new therapeutic options for PsA and a growing number of RCTs and LOS in PsA, it is important to understand how best to measure disease activity and its impact. GRAPPA/OMERACT have been playing a leading role in informing how best to assess PsA. The CASPAR criteria remain the cornerstone for classifying PsA patients for enrolment in RCTs and LOS. The PsA core domain set has been updated to guide the measurement of outcomes that are relevant to both clinicians and patients. It will be important to continue to standardize outcome measures for RCTs and LOS in PsA. Using OMERACT's updated methodology to generate the best evidence will be essential establishing consensus among various stakeholders. This standardized outcome measurement set will provide a standard for subsequent RCTs/LOS in PsA, as well as to assist clinicians and patients in understanding the best evidence for a particular treatment.

## Author contributions

YYL drafted the manuscript. All authors critically reviewed the content pertaining to their expertise in the topic.

### Conflict of interest statement

The authors declare that the research was conducted in the absence of any commercial or financial relationships that could be construed as a potential conflict of interest.
